# Epidemiology of Upper Limb Injuries in two major Brazilian Soccer Championships from 2016 to 2019

**DOI:** 10.1186/s40634-022-00560-1

**Published:** 2022-12-14

**Authors:** Ewerton Borges de Souza Lima, Gabriel Paris de Godoy, Guilherme Ladeira Osés, Paulo Henrique Schmidt Lara, Leandro Masini Ribeiro, Carlos Vicente Andreoli, Alberto de Castro Pochini, Paulo Santoro Belangero, Gustavo Gonçalves Arliani, Benno Ejnisman, Moisés Cohen

**Affiliations:** Department of Orthopaedics and Traumatology, Sports Traumatology Group, Discipline of Sports Medicine, Escola Paulista de Medicina, Federal University of São Paulo, R. Estado de Israel, 713 - Vila Clementino, São Paulo, SP Brazil

**Keywords:** Soccer, Epidemiology, Upper limb, Shoulder, Sports

## Abstract

**Purpose:**

To evaluate epidemiological data of upper limb injuries in professional athletes who participated in two major Brazilian soccer championships between 2016 and 2019.

**Methods:**

A prospective cohort study was conducted to evaluate the teams of two divisions over four seasons within the Brazilian Soccer Championship and the Paulista Soccer Championship. Clubs and their doctors were contacted to participate in the study and guided on the correct way to enter data via online platforms: Transfermarkt (Transfermarkt GmbH & Co. KG) and Survey Monkey (Momentive.AI). Demographic data, injury characteristics, and FIFA Incidence *Formula* were analyzed.

**Results:**

Overall, the study analyzed 3,828 matches and 126,357 hours of play. Upper limb injuries were registered 169 times, representing 6.8% of total injuries, with a FIFA incidence of 1.34. Most lesions occurred in forward players (21.3%), the shoulder exhibited the highest number of injuries (63.3%). The player’s position was related to the location on the field where the injury occurred (*p <* 0.001); however, there was no relationship between the type of injury and the location on the body (*p* > 0.001). The average time to return to play was 19.1 days (range 0–200 days) and it was longer for goalkeepers. The necessity of surgical treatment was statistically associated with additional time to return to play (*p <* 0.001).

**Conclusions:**

Shoulder injuries were the most frequent upper limb injury sustained during the two major Brazilian soccer championships. Forward players suffered the most upper limb injuries and goalkeepers experienced the longest time to return to play.

## Introduction

Soccer is the most popular sport globally, and Brazil has a worldwide representation, leading the sales of athletes abroad. The country was responsible for moving 223 million euros in early 2019 alone [1]. However, the sport is associated with a high risk of injury. Arliani et al. reported an incidence rate of 10 to 15 injuries per 1000 hours of play. Lower limb injuries are the most common, followed by upper limb injuries [2], which despite their lower incidence, can negatively impact the performance of athletes, lead to long periods of absence, and generate expenses for treatment and rehabilitation [3–5]. In that context, high injury rate impacts athletic performance and increases costs for soccer teams [6].

Soccer athletes are prone to upper limb injuries during games and training sessions, regardless of their playing position. Despite having negative implications to soccer clubs and athletes, these injuries are often overlooked and there are few researches about it. The FIFA 11+ program was a step towards the prevention of upper limb injuries, however it was directed mostly for goalkeepers [5, 7, 8]. Thus, new researches on the topic are important for the creation of new training protocols that cover the entire team of athletes.

To our knowledge, there are still no epidemiological studies that assess the incidence of upper limb injuries in Brazilian professional soccer and just a few that evaluate these cases worldwide. However, describing these injuries and their epidemiology is vital for injury prevention and the treatment of athletes. Furthermore, a better understanding of the epidemiology and incidence of upper limb injuries will help teams plan for such outcomes. Therefore, this study aimed to assess the incidence of upper limb injuries in professional athletes who participated in two major soccer championships in Brazil held between 2016 and 2019.

## Methods

This observational prospective and analytical cohort study evaluated the teams of two divisions over four seasons (2016 to 2019) within the Brazilian Soccer Championship (BSC) and the Paulista Soccer Championship (PSC). Clubs and their doctors were contacted to participate in the study and guided on the correct way to enter data via two online platforms: Transfermarkt (Transfermarkt GmbH & Co. KG) and SurveyMonkey (Momentive.AI). Club doctors were reminded monthly about completing the forms. Two forms were used; the first was completed shortly after each match and the second after the injured athlete returned to the sport. Data were included on players with upper limb injuries that occurred during championship games, without age restrictions.

Four BSC seasons were played during the study period, involving 20 clubs per division each year, with 38 matches played per season. As for the PSC, there were four seasons involving 16 to 20 clubs per division each year, with 17 to 22 matches played per season. In total, 15 seasons were analyzed between 2016 and 2019 (there was no data recorded for the 2019 season in the second division of the BSC), totaling 3,828 matches and 126,357 hours played.

Data relating to the players (age and position) and injuries (diagnosis, type, body location, laterality, field location, tests performed, need for surgery, time to return to play, and recurrence) were collected. In this study, injury was defined as any musculoskeletal complaint occurred during a match that caused the player to miss at least one training session or match. Injuries were classified into four types for analysis: contusion, fracture, musculotendinous injury, and dislocation based on doctor diagnosis. In addition, the incidence of lesions was analyzed according to the *FIFA Incidence Formula*: Incidence = (total injuries X 1000 hours) / exposure.

Excel 2016 (Microsoft Corporation, Redmond, WA, USA) was used for the descriptive statistical analysis of the quantitative variables. SPSS V20 (International Business Machines Corporation, Armonk, NY, USA) was used for the statistical inference of continuous variables. Shapiro Wilk test was used to establish normality. Qualitative variables were analyzed using a Chi-square test. ANOVA test was performed for multivariate analysis with a 95% CI and significance level of 5% (*p <* 0.05).

## Results

There were 7899 medical interventions on the field, of which 2486 resulted in the diagnosis of an injury. Of these, 169 were upper limb injuries, representing 6.8% of total injuries, with a FIFA incidence of 1.34. The age of the injured players ranged from 19 to 41 years, with a mean age of 26.7 years (SD = 4.62). The goalkeepers were the oldest, with a mean age of 29.7 years, and the youngest were the defenders with 25.4 years. Dislocations were the most prevalent type of injury, with a total of 96 cases (58.5%). Table 1 illustrates the incidence of upper limb injuries.

**Table 1:** Distribution of injury body location and type

Body Location	Contusion	Fracture	Musculotendinous Injury	Dislocation	Total
Forearm	2 (1.2%)	5 (3%)	0 (0%)	0 (0%)	7 (4.1%)
Arm	0 (0%)	1 (0.6%)	0 (0%)	0 (0%)	1 (0.6%)
Elbow	5 (3%)	0 (0%)	0 (0%)	7 (4.1%)	12 (7.1%)
Shoulder	21 (12.4%)	4 (2.4%)	4 (2.4%)	78 (46.2%)	107 (63.3%)
Hand and Wrist	16 (9.5%)	15 (8.9%)	0 (0%)	11 (6.5%)	42 (24.9%)
Total	44 (26%)	25 (14.8%)	4 (2.4%)	96 (56.8%)	169

The shoulder exhibited the highest number of injuries (*n* = 107), with a prevalence of 4.3% in relation to total injuries and 63.3% among upper limb injuries (*p <* 0.001). Injuries occurred on the right side in 52.7% of cases.

### Diagnosis

There were 14 different diagnoses of upper limb injuries (*p <* 0.001). The most frequent diagnosis was glenohumeral dislocation (23.7%, *n =* 40) followed by acromioclavicular dislocation (22.5%, *n =* 38). The other diagnoses were shoulder contusion (12.4%, *n =* 21), hand contusion (9.5%, *n =* 16), hand fracture (8.9%, *n =* 15), hand dislocation (6.5%, *n =* 11), elbow dislocation (4.1%, *n =* 7), elbow contusion (3%, *n =* 5), forearm fracture (3%, *n =* 5), rotator cuff injury (2.4%, *n =* 4), forearm contusion (1.2%, *n =* 2), clavicle fracture (1.2%, *n =* 2), proximal humerus fracture (1.2%, *n =* 2), and humeral diaphysis fracture (0.6%, *n =* 1).

### Player Positions and Body Location of Injuries

**Fig. 1:**
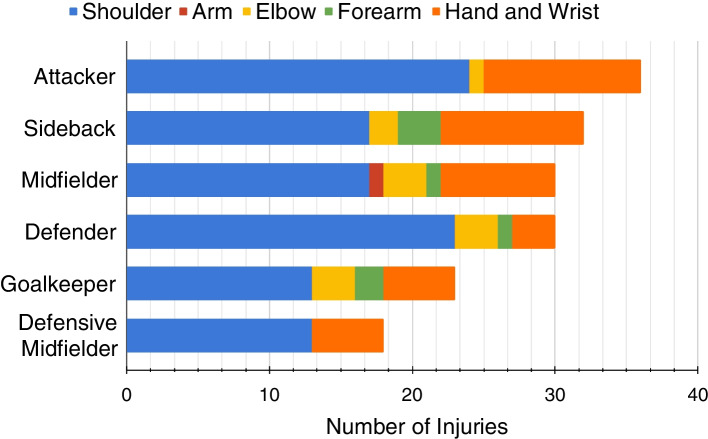
Upper limb injury incidence distributed by player position

In this study, the location on the body with the highest incidence of injuries, regardless of the player’s position, was the shoulder [63.3% (*n =* 107)]. Figure 1 illustrates the distribution of injury locations on the body according to the position of the player.

With respect to the position of the injured players, 21.3% were forward (*n =* 36), 18.9% side-backs (*n =* 32), 17.7% defenders (*n =* 30), 17.7% midfielders (*n =* 30), 13.6% goalkeepers (*n =* 23), and 10.6% defensive midfielders (*n =* 18).

Ten players (6%) experienced a recurrence of the injury (*p* = 0.037), but of the 169 athletes, this variable was only reported in 100 cases (59%). Relationship between recurrent injuries and surgical treatment was non-significant. Dislocations represented 100% of injury recurrences (*n =* 10). Injury recurrence was associated with diagnosis (*p* = 0.008), with glenohumeral and acromioclavicular dislocations representing 90% and 10% of cases, respectively.

The player’s position was related to the location on the field where the injury occurred (*p <* 0.001). Relationship between player position and type of injury or location on the body was non-significant. Detailed information and correlation to the FIFA incidence can be found in Table 2.

**Table 2:** Incidence of upper limb injuries by player position, field location, type of injury related to the FIFA incidence

Injury Characteristics		Attacker	Goalkeeper	Sideback	Midfielder	Defensive Midfielder	Defender	Total	FIFA Incidence
Field location	Attacking Area	13 (44.8%)	0 (0.0%)	2 (10.5%)	2 (16.7%)	2 (22.2%)	2 (9.5%)	21 (21.0%)	0.16
	Defending Area	3 (10.3%)	10 (100%)	6 (31.6%)	1 (8.3%)	3 (33.3%)	13 (61.9%)	36 (36.0%)	0.28
	Attacking Midfield	10 (34.5%)	0 (0.0%)	4 (21.1%)	6 (50.0%)	1 (11.1%)	0 (0.0%)	21 (21.0%)	0.16
	Defending Midfield	3 (10.3%)	0 (0.0%)	7 (36.8%)	3 (25.0%)	3 (33.3%)	6 (28.6%)	22 (22.0%)	0.17
Injury type	Contusion	11 (30.6%)	8 (34.8%)	6 (18.8%)	7 (23.3%)	7 (38.9%)	5 (16.7%)	44 (26.0%)	0.35
	Fracture	2 (5.6%)	6 (26.1%)	5 (15.6%)	6 (20.0%)	3 (16.7%)	3 (10.0%)	25 (14.8%)	0.20
	Musculotendinous Injury	0 (0.0%)	1 (4.3%)	0 (0.0%)	1 (3.3%)	0 (0.0%)	2 (6.7%)	4 (2.4%)	0.03
	Dislocation	23 (63.9%)	8 (34.8%)	21 (65.6%)	16 (53.3%)	8 (44.4%)	20 (66.7%)	96 (56.8%)	0.76
Body location	Forearm	0 (0.0%)	2 (8.7%)	3 (9.4%)	1 (3.3%)	0 (0.0%)	1 (3.3%)	7 (4.1%)	0.05
	Arm	0 (0.0%)	0 (0.0%)	0 (0.0%)	1 (3.3%)	0 (0.0%)	0 (0.0%)	1 (0.6%)	0.01
	Elbow	1 (2.8%)	3 (13.0%)	2 (6.3%)	3 (10.0%)	0 (0.0%)	3 (10.0%)	12 (7.1%)	0.09
	Shoulder	24 (66.7%)	13 (56.5%)	17 (53.1%)	17 (56.7%)	13 (72.2%)	23 (76.7%)	107 (63.3%)	0.84
	Hand and Wrist	11 (30.6%)	5 (21.7%)	10 (31.3%)	8 (26.7%)	5 (27.8%)	3 (10.0%)	42 (24.9%)	0.33
FIFA Incidence		0.28	0.18	0.25	0.24	0.14	0.24	1.34	

### Diagnostic imaging test

Completed complementary examinations were recorded in 153 of the 169 injury cases (90.5%). Of these, 33 (21.5%) players did not undergo any type of diagnostic imaging test, 126 (78.4%) completed at least one type of diagnostic imaging test (x-ray, ultrasound, magnetic resonance, computerized tomography, others), 11 (7%) underwent two tests, and 7 (4.5%) completed three of these examinations.

### Time to Return to Play

The average time to return to play (TRP) was 19.1 days, ranging from 0 to 200 days. Sixteen (9.4%) injured athletes requires surgical intervention, resulting in a TRP that ranged from 21 to 200 days. The necessity of surgical treatment was statistically associates with additional TRP (*P <* 0.001). Relationship between TRP and player position was non-significant.

The injuries were classified by severity according to TRP, as suggested by Ekstrand et al. [9]: mild (< 4 days), minor (4–7 days), moderate (8–28 days), major (4–8 weeks), and severe (> 8 weeks) injuries. There was no record of TRP in sixteen cases, making it impossible to classify their severity. Data regarding the severity of the injury in relation to the affected body part is shown in Table 3.

**Table 3:** Relation between severity of injury and body location

Body Location	Mild	Minor	Moderate	Major	Severe	Total
Shoulder	29 (51.8%)	24 (62%)	25 (67.5%)	8 (57%)	9 (56.2%)	95
Arm	0 (0%)	0 (0%)	0 (0%)	0 (0%)	1 (6.3%)	1
Elbow	5 (8.9%)	2 (5.4%)	2 (5.4%)	2 (14.2%)	0 (0%)	11
Forearm	2 (3.6%)	0 (0%)	1 (2.7%)	0 (0%)	4 (25%)	7
Hand and Wrist	20 (35.7%)	4 (11%)	9 (24.3%)	4 (28.5%)	2 (12.5%)	39
Total	56 (33.1%)	36 (21.3%)	37 (21.8%)	14 (8.2%)	16 (9.4%)	153

## Discussion

The most important findings of this study were that upper limb injuries represented 6.8% of total injuries and the shoulder represented the most injured location. In addition to it, glenohumeral and acromioclavicular dislocation were the most common type of lesion and responsible for all the recurrent lesions. Injuries in soccer have a significant impact on the health and performance of players and affect the involved clubs and athletes financially [3–5]. For instance, our results, showed players submitted to surgical treatment presented longer time to return to play.

In general, the incidence of injuries in both championships was 19.6 per 1000 hours played. For upper limb injuries, this incidence was 1.33 per 1000 hours played. A previous study in the English women’s and men’s soccer Leagues revealed an incidence of 31.1 general injuries per 1000 hours of play [10]. Jan Ekstrand et al. reported an incidence of only 0.23 per 1000 hours played for upper limb injuries when monitoring the European soccer league [9]. The differences in results may be attributed to the match particularities in each league, duration of the season, sample size, and the quality of data regarding injuries during the tournaments [9, 11].

The results showed an upper limb injury incidence of 6.8% in relation to total injuries. Shoulder injuries accounted for 4.3% of the total injuries and 63.3% of upper limb injuries, corroborating similar findings in previous studies [4, 12]. Although upper limb injuries are less frequent in soccer players, the sport has evolved into a high-speed game with tactical plays involving more significant physical contact, which may predispose to traumatic injuries [8, 13–16].

The increasing prevalence of shoulder injuries has become a health problem for athletes, which led to the development of the FIFA 11+ program to prevent them [8, 16]. The program was developed with a focus on goalkeepers, as previous studies has shown that players in this position have a higher incidence of shoulder injuries [9, 17–19]. Our study showed few injuries in goalkeepers, but this may be due to sample bias. In addition, although shoulder injuries are the most frequent upper limb injuries in soccer, other injuries occur and negatively impact players and clubs [17, 19, 20]. Thus, additional studies are needed, focusing on upper limb injuries in soccer athletes, to better understand the changes in the sport and the profile of the injuries [19].

Forward and players in defending positions (defender, defensive midfielder and side-backs) had higher rates of upper limb injuries and presented with a higher injury risk. These results corroborate previous studies in which changes in the direction of play, speed, and strength cause attacking and defending players to collide with opponents, favouring the occurrence of injuries [13–15]. However, other studies in the literature report that the goalkeeper has the greatest potential to suffer an upper limb injury [9, 18, 19]. Differences in the results may be due to sample size differences between studies, game style and the tournament characteristics.

In this study, 66.9% of upper limb injuries resulted in a TRP greater than four days, with an average of 19.1. Considering that each season had an average duration of 154.9 days, players who suffered injuries lost approximately 12% of the season. In cases that required surgery, the lost time lasted the remainder of the season. A previous study similarly reported that the average TRP of players due to injuries was 12% off the season; however, the primary injuries affected the lower limbs [21].

The mean TRP was 23 days per 1000 hours played. Thus, the results of this study showed a longer mean TRP for upper limb injuries than lower limb injuries, according to the literature [9, 17, 18]. We believe this is due to soccer being a primarily lower limb sport, making injuries more common to this part of the body, even though most of them are mild. On the other side, upper limb injuries are less common, but often caused by more intense traumas, leading to longer TRP. Therefore, measures to prevent upper limb injuries need to be developed, and it is recommended that athletes strengthen this part of their body.

Previous studies in Brazilian Championships reported a rate of 11.9% for surgical interventions compared to 9.4% in this study [22]. Nevertheless, the TRP of the players who needed surgical intervention exceeded that of those who did not need it, leading to more significant implications for clubs and athletes.

The TRP players experience due to an injury can impact the performance of their clubs as well as the athlete’s performance. The severity of the injury and the need for surgery were both associated with prolonged lost time [3, 23]. In addition, the lost time of a player due to an upper limb injury increases club financial expenses [18]. These results bring awareness of how beneficial prevention of upper limb injuries are to mitigate lost time consequences.

Several studies report on the mechanisms of injuries in soccer and how to prevent them, but most focus on lower limb injuries [7, 24]. In general, injury prevention in soccer reduces the incidence of injuries and health costs [5, 25]. However, more research is needed to understand the mechanisms of upper limb injuries in soccer and better assist coaches, doctors, and athletes in preventing these injuries [26].

This study’s primary limitation is the possible data collection bias, as it is inherent in epidemiological studies, since the club physicians were responsible for entering data into the platform. Nevertheless, it is a four-year-long prospective study, during this period, there were some changes of physicians at the clubs. To reduce bias of data collection, every physician was trained how to fill the survey and every time the club physician was substituted, we were informed, and the new physician was trained too. Nevertheless, it was possible to collect novel data of two major Brazilian soccer championships regarding upper limb injuries. Another limitation is that there is no consensus of the definition of injury in the literature, making it difficult to compare our results with other studies. In addition, data was not collected for injuries that occurred during the training sections.

## Conclusion

Shoulder injuries were the most frequent upper limb injury during two major Brazilian soccer championships. Dislocations were the most common type of injury and were associated with episodes of recurrence. Glenohumeral dislocations were the most prevalent diagnosis, primarily affecting the defenders. Forward players suffered the most upper limb injuries, and the goalkeepers were the ones who had the most lost time. Injuries requiring surgical treatment were associated with additional lost time.

## Data Availability

The datasets used and/or analysed during the current study are available from the corresponding author on reasonable request.

## Abbreviations

BSC: Brazilian Soccer Championship
PSC: Paulista Soccer Championship
FIFA: Fédération Internationale de Football Association
TRP: Time to return to play
